# A novel, sequencing-free strategy for the functional characterization of *Taenia solium* proteomic fingerprint

**DOI:** 10.1371/journal.pntd.0009104

**Published:** 2021-02-18

**Authors:** Sandra Gomez-Fuentes, Sarah Hernández-de la Fuente, Valeria Morales-Ruiz, Dina López-Recinos, Adrián Guevara-Salinas, María Cristina Parada-Colin, Clara Espitia, Adrián Ochoa-Leyva, Filiberto Sánchez, Nelly Villalobos, Asiel Arce-Sillas, Marisela Hernández, Silvia Ivonne Mora, Gladis Fragoso, Edda Sciutto, Laura Adalid-Peralta

**Affiliations:** 1 Unidad Periférica de Neuroinflamación para el estudio de patologías neurológicas del Instituto de Investigaciones Biomédicas en el Instituto Nacional de Neurología y Neurocirugía, México, México; 2 Departamento de Inmunología, Instituto de Investigaciones Biomédicas, Universidad Nacional Autónoma de México, México, México; 3 Instituto de Biotecnología, Universidad Nacional Autónoma de México, Chamilpa, Cuernavaca, Morelos; 4 Facultad de Medicina Veterinaria y Zootecnia, Universidad Nacional Autónoma de México, Universidad Nacional Autónoma de México, México, México; 5 Instituto Nacional de Neurología y Neurocirugía, La Fama, México, México; Wellcome Sanger Institute, UNITED KINGDOM

## Abstract

The flatworm *Taenia solium* causes human and pig cysticercosis. When cysticerci are established in the human central nervous system, they cause neurocysticercosis, a potentially fatal disease. Neurocysticercosis is a persisting public health problem in rural regions of Mexico and other developing countries of Latin America, Asia, and Africa, where the infection is endemic. The great variability observed in the phenotypic and genotypic traits of cysticerci result in a great heterogeneity in the patterns of molecules secreted by them within their host.

This work is aimed to identify and characterize cysticercal secretion proteins of *T*. *solium* cysticerci obtained from 5 naturally infected pigs from Guerrero, Mexico, using 2D-PAGE proteomic analysis. The isoelectric point (IP) and molecular weight (MW) of the spots were identified using the software ImageMaster 2D Platinum v.7.0. Since most secreted proteins are impossible to identify by mass spectrometry (MS) due to their low concentration in the sample, a novel strategy to predict their sequence was applied. In total, 108 conserved and 186 differential proteins were identified in five cysticercus cultures. Interestingly, we predicted the sequence of 14 proteins that were common in four out of five cysticercus cultures, which could be used to design vaccines or diagnostic methods for neurocysticercosis. A functional characterization of all sequences was performed using the algorithms SecretomeP, SignalP, and BlastKOALA. We found a possible link between signal transduction pathways in parasite cells and human cancer due to deregulation in signal transduction pathways. Bioinformatics analysis also demonstrated that the parasite release proteins by an exosome-like mechanism, which could be of biological interest.

## Author summary

Human neurocysticercosis is a major neglected tropical disease caused by *Taenia solium*. As part of their natural metabolism, cysticerci release molecules into their microenvironment within the host. Secreted molecules could be useful in the design of vaccine candidates or diagnostic methods, or as therapeutic targets. Unfortunately, most secreted proteins cannot be identified by mass spectrometry sequencing due to their low concentration in the sample. In this study, we introduce a novel strategy to predict the sequence of *T*. *solium* secreted proteins using 2D-PAGE proteomic analysis and the *T*. *solium* genome. This approach allowed us to identify proteins with potential applications in the diagnosis and/or prevention of neurocysticercosis.

## Introduction

Cysticercosis is a parasitic disease that affects humans and swine. It is caused by the establishment of the larval form of *Taenia solium* in skeletal muscle and brain tissues. When cysticerci are established in the central nervous system of the host, they cause neurocysticercosis (NC). This potentially life-threatening disease is endemic in rural areas of Latin America, Southeast Asia (India, China, and Nepal) and sub-Saharan Africa, where poverty prevails and hygiene is poor [1]. Unfortunately, human migration from endemic zones has reintroduced NC in Europe, Canada, and especially the United States, where more than 5000 infected patients were reported in the last decade [2,3].

Cysticerci produce and secrete several molecules as a result of their metabolism, including digestive enzymes, extracellular proteinases, cytokine-like, chemokine-like, and hormone-like proteins, and others. Secreted proteins (ESPs) could provide us with important information on the host-parasite relationship [4–6]. ESPs have also been involved in key biological processes like adhesion, migration, cell-cell communication, differentiation, proliferation, morphogenesis, survival, defense, virulence, and immune response [7].

Thus, considerable attention has been paid to parasite-secreted proteins in various infectious diseases, searching for biomarkers to detect the presence of a parasite and/or the status of the infection [8–11]. The identification of secretion proteins involved in the pathogenesis of parasitic infections could lead to the discovery of therapeutic targets, as well as useful biomarkers in diagnostic assays [4].

Victor et al. (2012) first reported and characterized *Taenia solium* secretion proteins. These authors used a database of secretion proteins from closely related helminths and the expressed sequence tags combined with BLAST and a mapping of super contigs in the genome of *Echinococcus granulosus*, taking advantage of its biological closeness with *T*. *solium*. While this strategy allowed to outline the possible secretome of *T*. *solium*, it proved to have great limitations, and only 76 proteins, mostly involved in parasite survival, were identified as ESP. Among these proteins, only 27 had been previously described in *T*. *solium*; 32 proteins were identified as probably of *T*. *solium* origin by their homology to sequences found in other helminths, and 17 proteins were determined as produced by the host (*Sus scrofa*) [6].

In 2015, our research team published the complete *T*. *solium* secretome, obtained directly from the genome of the helminth; in total, 838 proteins were identified and characterized by their function and antigenicity [12]. The increasing availability complete genome sequences, that allowed us to systematically examine genetic data and provided us with a complete list of promising biological markers instead of a limited set of candidates, demonstrating the validity of bioinformatics-based approaches to predict a secretome [12].

This secretome was validated using a TMT-multiplexed strategy [13]. These authors identified 4200 proteins in the *T*. *solium* cysticercus proteome, the most extensive intermixing of host- and parasite-derived proteins reported for tapeworm infections. Their data set was then compared with previous proteomic reports for other helminths [14–16]. Additionally, they used our report on *T*. *solium* secretome [12] to identify 167 excretory/secretory proteins in their data set. Using various algorithms, those authors select 9 antigenic proteins and used them to design a diagnostic assay (ELISA) to detect porcine cysticercosis [13].

Bioinformatic analysis has proved to be useful for protein identification and characterization. Three main research approaches have been reported. First, transcriptomic data (mRNA) can be used for the computational prediction of the signal peptide in genomic sequences and characterize them by computational tools [4]. Second, EST tags (EST2secretome) can be generated in a bioinformatic approach, where the signal peptide is identified, the sequences showing transmembrane motifs are eliminated, and ontological analyses are performed to infer functions. The mapping relies on known domains and metabolic pathways, as well as on the search for homologs [17].

In the third strategy, a proteomic analysis is conducted, based on experimental data from two-dimensional gels and subsequent MS sequencing. While the latter is the most useful method when no genomic data is available [4], it is often the case that the products of interest are only a small fraction of the proteins in a complex mixture [18]. The high variability in protein expression levels severely limits the ability of 2DPAGE-MS to identify low-abundance proteins [19]. Although 2DPAGE-MS is a powerful, mature, and sensitive technique, it is often not optimal for the analysis of a complete proteome. It has been shown that only 40% of the differentially expressed proteins identified were sufficiently abundant to be identified by MS [4,20].

Thus, novel techniques that allow large-scale protein identification from a complex biological source, regardless of their relative expression, are much needed [20]. A viable alternative is bioinformatics analysis based on proteomics data.

Considering this, we designed a new strategy to characterize the individual protein profile of different cysticercus culture supernatants. First, a proteomic profile was determined for each culture by 2D-PAGE; then, secretome data were used to predict protein sequences [12]. This strategy allowed us to identify conserved and differential proteins between cultures, as well as to elucidate functions of secreted proteins and their possible therapeutic and/or diagnostic applications.

## Materials and methods

### Ethics statement

The project was approved by the Scientific and Ethics committees of the Institute National of Neurology and Neurosurgery “Manuel Velasco Suarez”, Mexico, permit number 42/15.

### Proteomic analysis

#### Parasites

**Fig 1:**
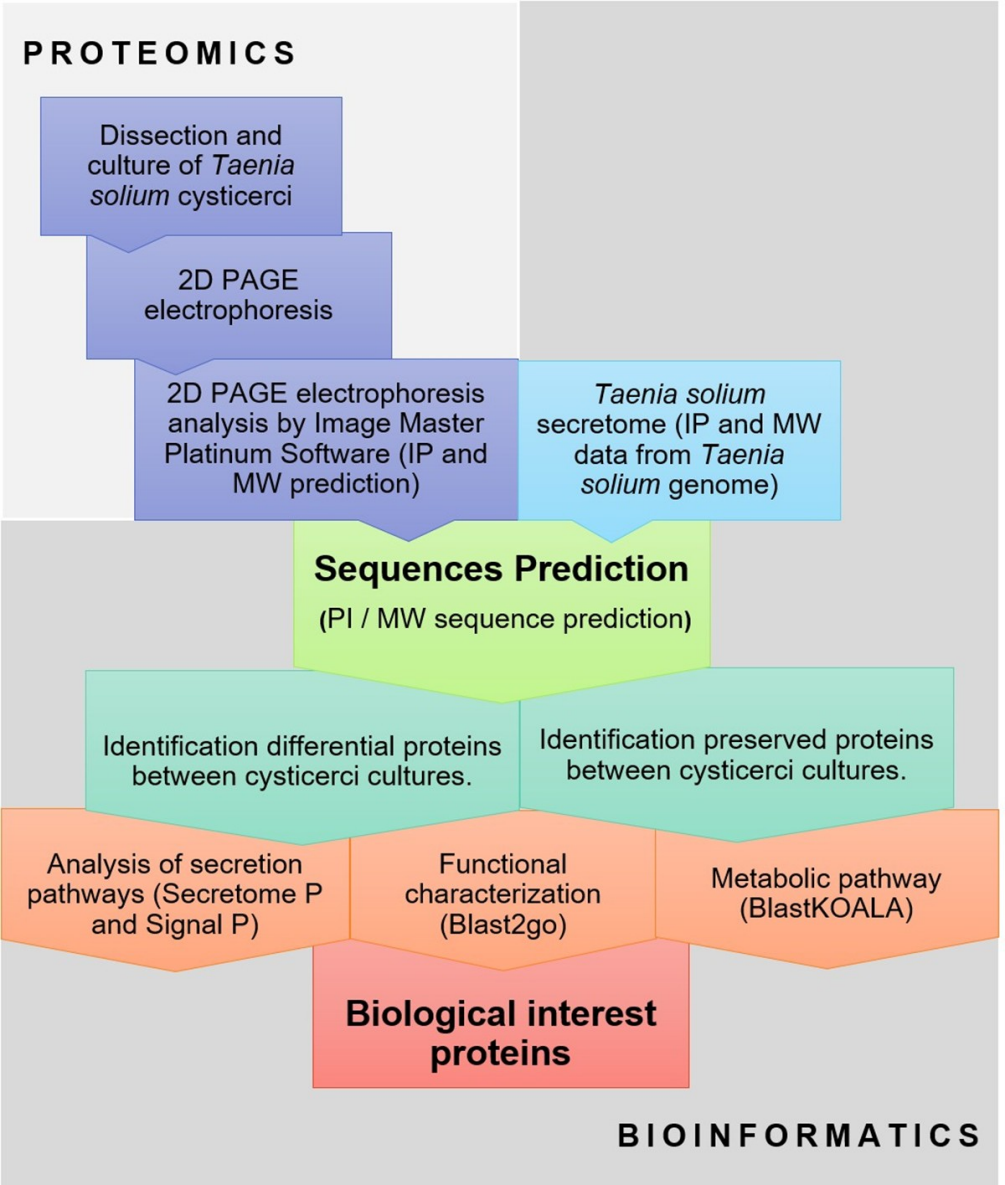
Experimental strategy to identify proteins resolved in 2D-PAGE gels by comparation with *Taenia solium* secretome. Isoelectric point (IP) and molecular weight (MW).

Viable *T*. *solium* cysticerci were recovered from five naturally infected pigs from the state of Guerrero, an endemic region for taeniasis/cysticercosis in Mexico. The pigs were euthanized in accordance with all applicable ethical guidelines. Cysticerci were individually excised from muscle tissue and placed in isotonic saline solution (ISS) (Fig 1).

#### Cysticercus culture

*T*. *solium* cysticerci were washed three times with sterile ISS. In total, 900 parasites were cultured in 900 ml of RPMI 1640 (Gibco, Thermo Fisher Scientific, Waltham, MA) supplemented with 10% FCS (Gibco) and Pen-strep antibiotics (Gibco) for 3 days at 37°C under 5% CO_2_. The medium was replaced every 24 h to remove host components [21] On day 4, RPMI was replaced by DMEM (Gibco) supplemented with L-glutamine (Gibco) and antibiotics (Gibco). The medium was collected every 48 h and replaced by fresh DMEM. The cultures were kept at 37°C under 5% CO_2_ for 21 days (Fig 1).

#### Protein purification

The collected supernatants were pooled and centrifuged at 1200 rpm for 10 min. Then, the supernatants were dialyzed against distilled water through a Spectra 3 membrane with a 3.5-kDa pore size (Spectra/Por, Rancho Dominguez, CA) at 4°C for five days. Once dialyzed, 300 ml of dialyzed supernatants were freeze-dried at −45°C and 133 ×10^−3^ mBar for 48 h in a Freezone 4.5 Dry Freeze system (Labconco, Kansas City, MO). The resulting powder was reconstituted with ultrapure DNase/RNase-free distilled water (Invitrogen, Carlsbad, CA) in a 1:10,000 ratio. Protein concentration was determined with the 2-D Quant Kit (GE Healthcare, Chicago, IL) following the manufacturer’s protocol (Fig 1).

#### 2D PAGE analysis

Isoelectric focusing was performed as described elsewhere, with some modifications [22]. The sample was desalted in an Illustra NAP 5 column (GE healthcare), and the proteins were then concentrated by freeze-drying (Labconco, Kansas City, MO) and precipitated by adding TCA-DOC-acetone (2% sodium deoxycholate (DOC) to a final 0.02% concentration, 100% trichloroacetic acid (TCA) to a final 10%, 200 μl of ice-cold acetone). The protein pellet was resuspended, and the final volume was adjusted to 125 μl with rehydration buffer (8 M urea, 2% CHAPS, 0.5% IPG buffer pH 4–7, and 20 mM DTT). Then, the sample was applied on Immobiline DryStrip pH 3–10, 7-cm linear-gradient strips (GE healthcare) for 16 h to dehydrate at room temperature, following the manufacturer’s instructions. Focusing was performed in an Ettan IPGphor 3 Isoelectric Focusing System (GE healthcare) starting at 500 V (for 5 h), increasing potential to 4500 V (90 min) and finally to 14,000 V. After focusing, the strips were equilibrated for 20 min in sample buffer (2% SDS, 50 mM Tris-HCl pH 8.8, 6 M urea, 30% glycerol, 0.002% bromophenol blue, and 0.5% DTT). The strips were then transferred onto 4.5–15% SDS-PAGE. After electrophoresis, the gels were stained with Coomassie Brilliant Blue R-250 (Bio-Rad Laboratories, Hercules, CA).

Isoelectric point (IP) and molecular weight (MW) were determined for all spots in the two-dimensional gels with the software ImageMaster 2D Platinum v.7.0 (GE Healthcare). These data were compared with the *T*. *solium* secretome (Fig 1). A protein in *T*. *solium* secretome with matching IP and MW was selected for each spot in the gels [12].

### Bioinformatics strategy

Predicted IP and MW values for the *T*. *solium* secretome were obtained from the *T*. *solium* genome database (Fig 1). *T*. *solium* genome is available on request from the authors of the original genomic sequencing article [23].

### Tandem mass spectrometry (LC/ESI-MS/MS)

Six large well-defined spots were sequenced by LC/ESI-MS/MS as described previously [24]. The spots were excised from Coomassie blue-stained gels and digested with modified porcine trypsin (Promega, Madison, WI). Mass spectrometry analysis was performed in a 3200 QTRAP System (Applied Biosystems/MDS Sciex, Canada) equipped with a nanoelectrospray source and a nanoflow LC system (Agilent 1100 Nano Pump, Waldbronn, Germany), coupled to an Acquity Ultra Performance LC system (Waters Corporations, Milford, MA). For data interpretation and protein identification, MS/MS spectrum datasets were searched using the MASCOT search algorithm v.1.6b9, Matrix Science, London, UK, available at http://www.matrixscience.com). A search was conducted in the genome of *T*. *solium* (available on request [23]) and in the National Center for Biotechnology Information non-redundant database (NCBInr).

#### Secretion pathways

Secretion pathways were determined for the proteins identified in our sequence-based IP-MW model with the tools SignalP v.5 [25] and SecretomeP (v.2.0) [26]. SignalP was used to predict classical ESPs (signal-peptide protein secretion pathway), set for eukaryotic organisms and with a position limit every 70 truncation residues. SecretomeP was used to predict non-classical ESPs (ER/Golgi-independent protein secretion pathway), using the default values for mammals (Fig 1).

#### Functional characterization

To determine the possible functions of the proteins in the two-dimensional gels identified by sequence prediction in our IP-MW model, functional annotation for the *T*. *solium* genome was performed with the servers Blast2Go and BlastKOALA [27].

## Results

### Proteomic analysis

#### Identification of conserved and differential protein spots

*T*. *solium* cysticercal ESPs were obtained and characterized by 2D-PAGE gels. The proteins were separated according to IP and MW as discrete spots. Each cysticercus culture showed a unique protein profile in terms of the number and location of spots on the gel; however, most culture samples share a common distribution pattern. Two distinct regions can be observed in all 2D-PAGE gels: a low-MW region (< 20 kDa) was protein-enriched, irrespective of IP values, while another enriched region is found at IP values between 5 and 8, regardless of MW.

**Fig 2:**
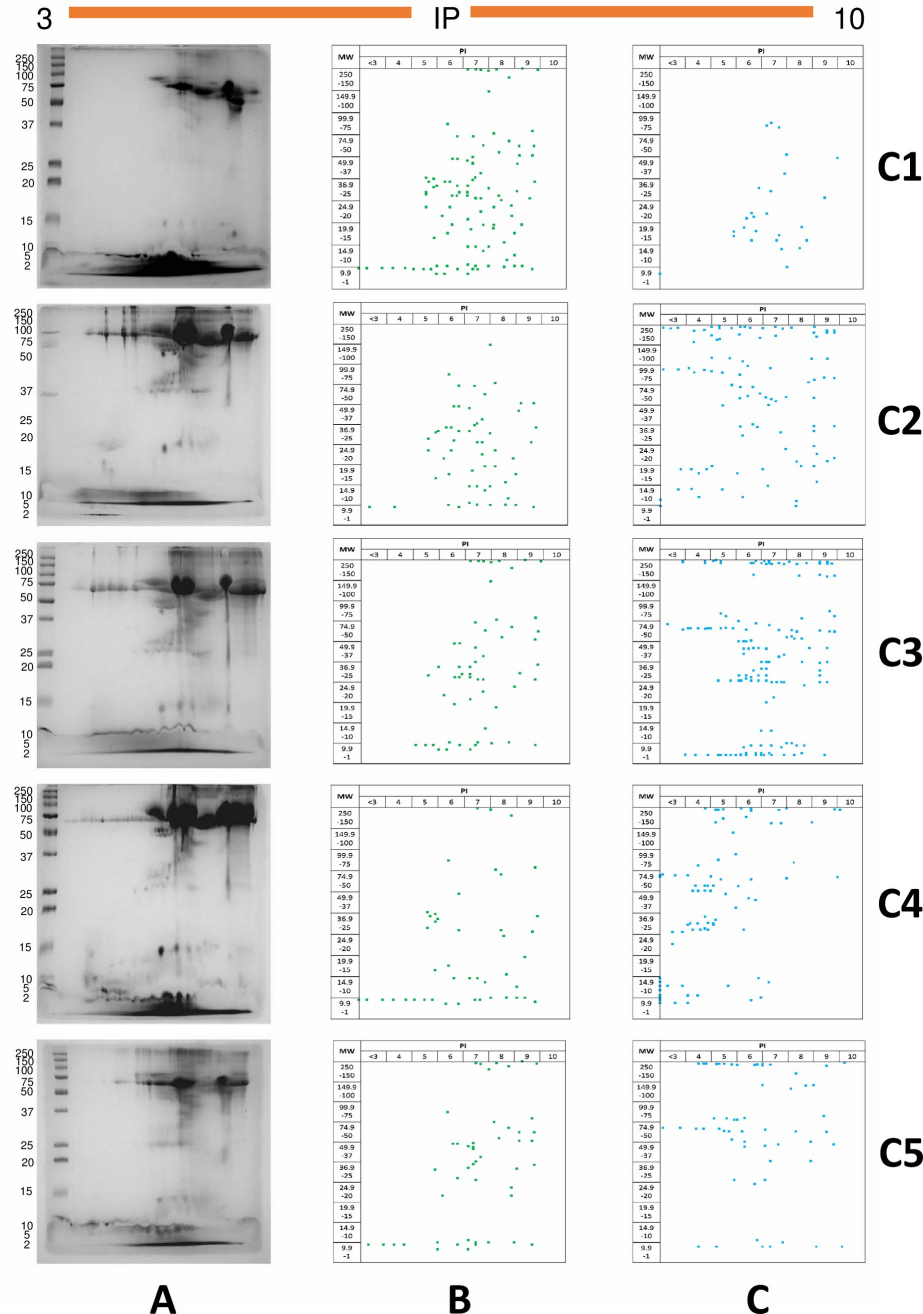
Spot identification in *Taenia solium* cysticercus culture 2D-PAGE gels. (A) 2D PAGE. (B) Conserved spots are marked in green (a conserved spot is one that is found in at least two different cultures). (C) Differential spots are marked in blue (a differential spot is one that is found in only one culture); isoelectric point (IP), culture 1 (C1), culture 2 (C2), culture 3 (C3), culture 4 (C4), and culture 5 (C5).

Fig 2A shows the proteomic fingerprint of cysticercus cultures. The number of spots ranged from 105 in culture 5 to 153 in culture 2. IP and MW values were determined for each spot with the software ImageMaster 2D Platinum v.7.0.

Spots in gels were classified into conserved and differential, according to IP and MW data. A spot was regarded as conserved when identical IP and MW values were determined for it in at least two cultures (Fig 2B), and a spot was considered as differential when no matching IP and MW values were found in other cultures (Fig 2C). Interestingly, at least one-third of protein spots were conserved among cysticercus cultures.

**Fig 3:**
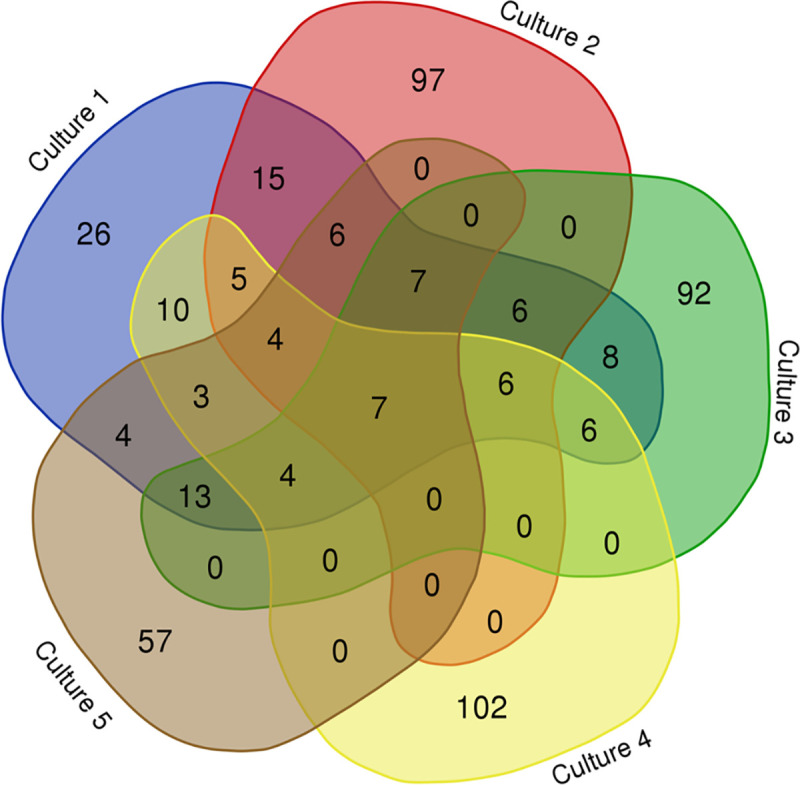
Protein interaction map for the 5 cysticercus cultures (culture 1 (C1), culture 2 (C2), culture 3 (C3), culture 4 (C4), and culture 5 (C5)). The graph was created with the bioinformatics tool Calculate and Draw Custom Venn diagrams (http://bioinformatics.psb.ugent.be/webtools/Venn/).

A protein interaction map was designed to establish the segregation of spots in 2D PAGE gels and determine the number of conserved and differential spots for each culture (Fig 3). As shown, 7 matching spots were conserved in all cultures, while 6 matching spots were found in C1, C2, C3, and C4; 6 matching spots were found in C1, C2, and C3. On the other hand, 4 matching spots were found in C1, C2, C4, and C5. Four matching spots were found in C1, C3, C4, and C5 (Fig 3). This map revealed the interrelations among cysticercus cultures and provided valuable information on proteins.

### Protein sequence prediction

In a previous work, our research group determined the *T*. *solium* secretome [12]. Protein sequences in the secretome were also known after the genome of *T*. *solium* was published [23]. IP and MW values for 838 proteins were retrieved from this database and used in this work.

We were interested in identifying the proteins found in the proteomic analysis of *T*. *solium* cysticercus cultures by 2D-PAGE gels, based on IP and MW data. To predict the protein sequence for each spot, IP and MW experimental values were compared with those values predicted for the proteins in the *T*. *solium* secretome. This strategy allowed us to determine the sequence of 455 proteins (including redundant proteins in the cultures) that, due to their low concentration in the sample, cannot be identified by MS sequencing. The S1–S5 Tables show the proteins identified in *T*. *solium* cysticercus cultures.

To validate our protein sequence prediction method, six high-expression protein spots were sequenced by MS spectrometry. As shown in the S6 Table, the same IP and MW were determined by the two methods.

**Fig 4:**
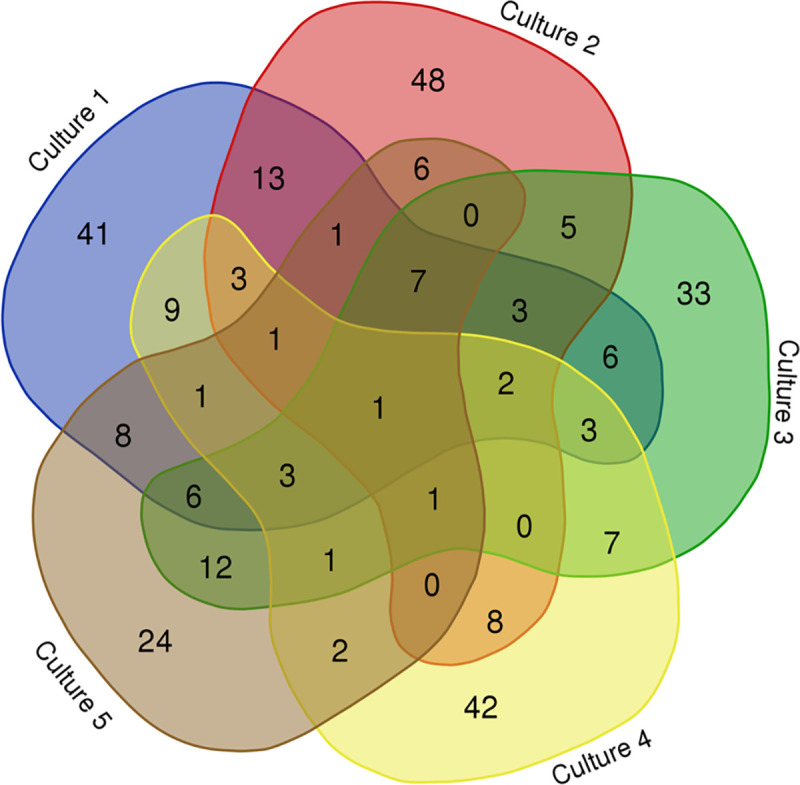
Protein interaction map for the 5 cysticercus cultures that matched with the *Taenia solium* secretome (culture 1 (C1), culture 2 (C2), culture 3 (C3), culture 4 (C4) and culture 5 (C5)). The graph was created with the bioinformatics tool Calculate and Draw Custom Venn diagrams (http://bioinformatics.psb.ugent.be/webtools/Venn/).

This strategy was applied to conserved and differential spots in each culture (S1–S5 Tables). Additionally, a theoretical two-dimensional gel was built with IP and MW values as plane coordinates of the spots in the cultures that matched *T*. *solium* secretome. As expected, a greater number of proteins matching the secretome were found in low-MW regions (< 20 kDa) (S1 Fig). In addition, a protein interaction map was created to predict the segregation of spots. As shown in Fig 4, one protein was conserved in all cultures in the interaction map, while 14 proteins were conserved in four of the five cultures (Fig 4).

**Fig 5:**
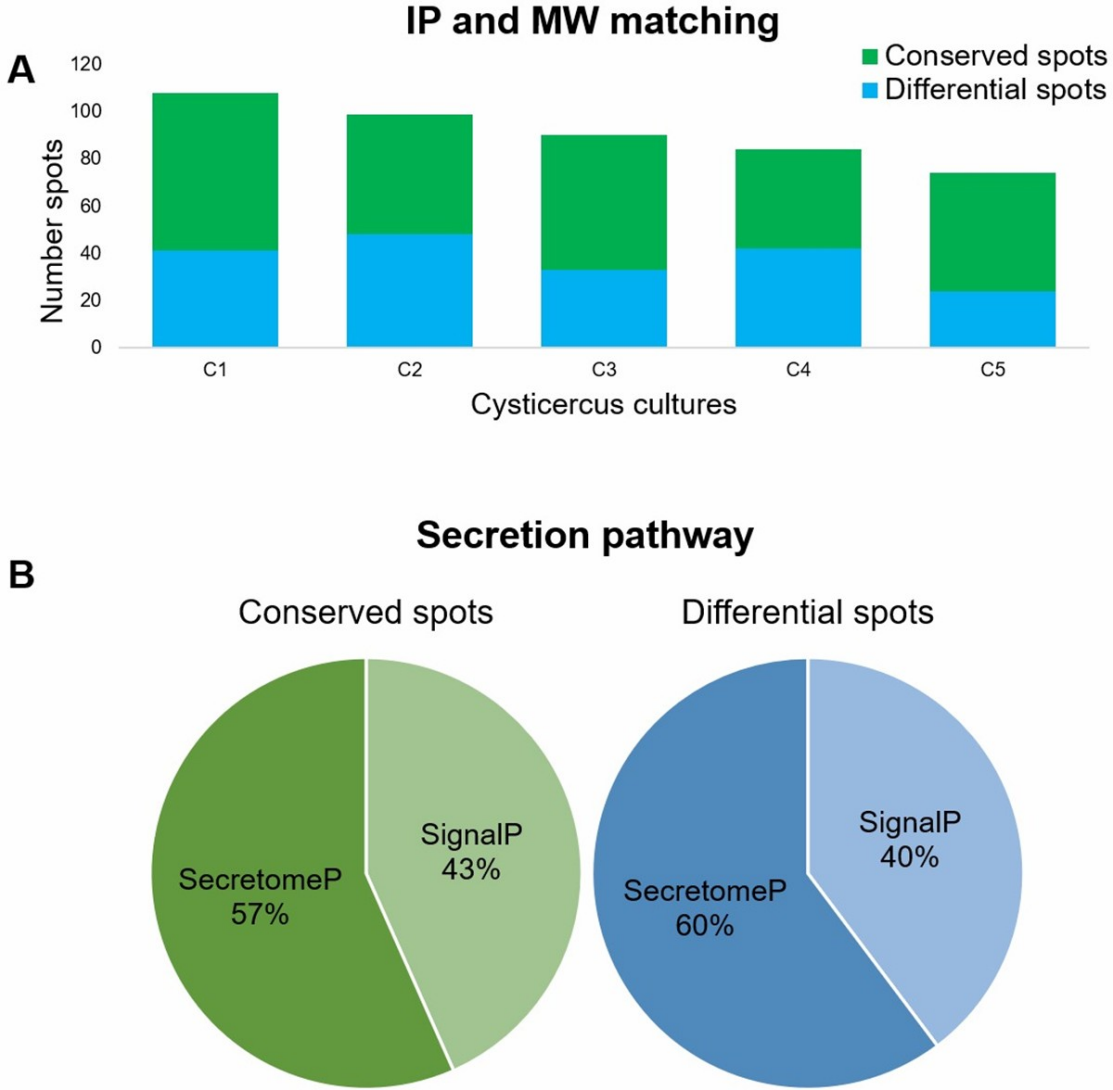
Analysis of 2D-PAGE. (A) Identification of conserved and differential spots in 2D gels. Conserved and differential spots in cysticercus cultures matching the *Taenia solium* secretome. (B) Secretion pathway for conserved and differential proteins matching the *Taenia solium* secretome.

As shown in Fig 5A, each culture showed a unique proteomic profile. This is probably due to genotypic differences in cysticerci and to their environmental history. It is important to consider that cysticerci were excised from naturally infected female pigs from Guerrero, an endemic region in Mexico for taeniasis/cysticercosis.

Various secretion routes have been described for proteins, depending on their fate in the cell. Considering this, the secretion pathway for the *T*. *solium* proteins identified was assessed. Secretion pathways were mapped with two tools widely used for this purpose, SignalP and SecretomeP. As seen in Fig 5B, most proteins in our cultures were secreted by a non-classical (ER/Golgi-independent) pathway.

### Functional analysis of proteomic profile

**Fig 6:**
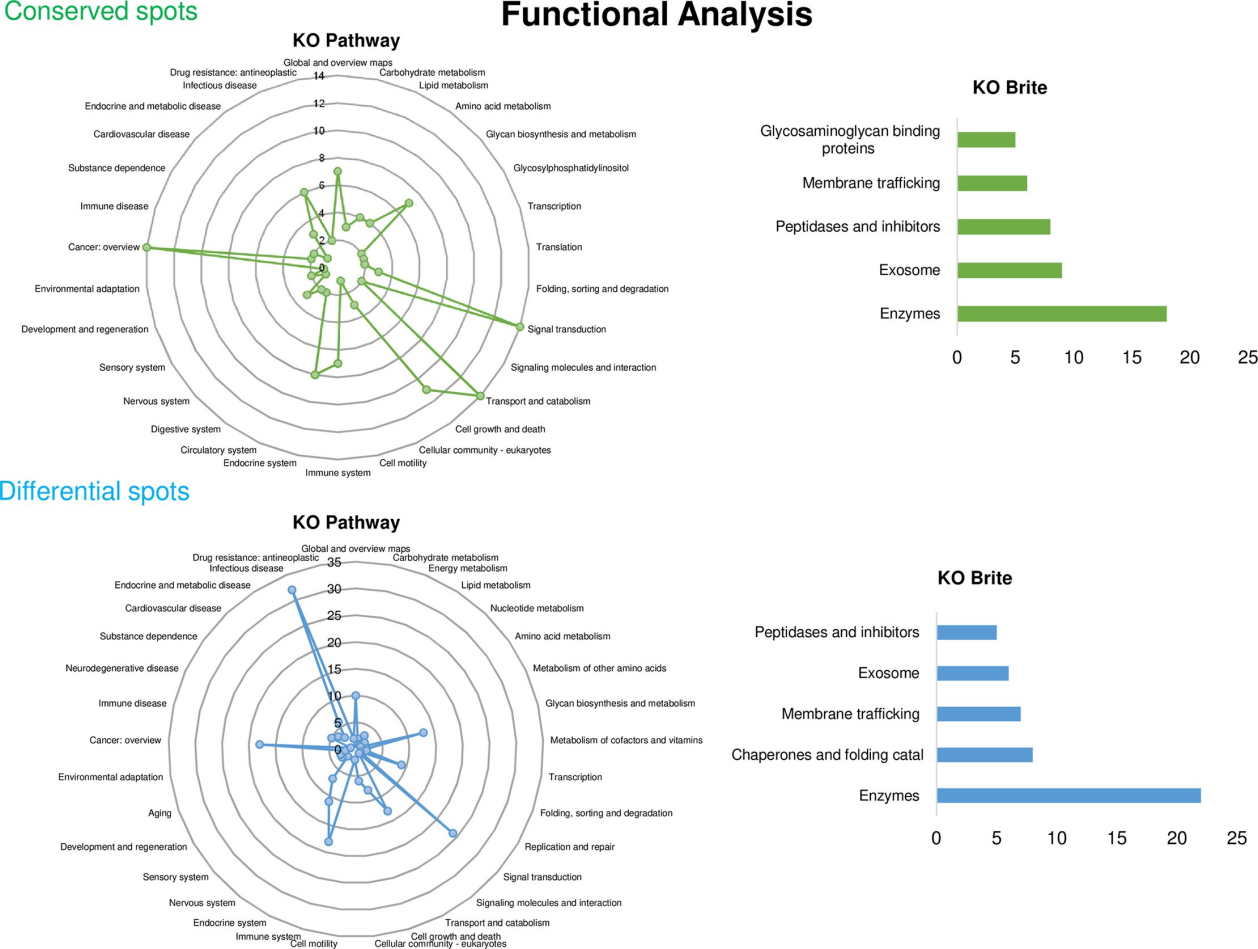
Functional characterization from conserved and differential proteins that match with the *Taenia solium* secretome. A) KO PATHWAY B) KO BRITE top 5.

To predict the functions of the proteins in cysticercus cultures whose sequence was identified, the annotation tool BlastKOALA, which uses the KEGG database to link biological functions, was used on *T*. *solium* genome. In total, 39.8% of conserved sequences had some annotation. Fourteen proteins were involved in signal transduction, 14 were involved in cancer development, 14 were involved in transport and catabolism, and 6 were involved in infectious pathologies, linked with some KO pathway. On the other hand, 18 proteins were annotated with enzymatic function and 9 were involved in exosomes when the annotation tool KO Brite was used (Fig 6). With respect to differential protein spots, 27.4% of sequences were annotated. In total, 32 proteins were involved in infectious pathologies, 24 were involved in signal transduction, 18 were involved in the immune response, 18 were involved in cancer development, 13 were involved in biosynthesis and glycan metabolism, and 13 were involved in transport and catabolism, annotated with KO pathway. The analysis with the tool KO Brite showed 22 proteins involved in enzymatic functions, 8 proteins acting as chaperones and folding catalysts, 6 proteins involved in exosome production, and 7 involved in membrane traffic (Fig 6).

## Discussion

Massive genome sequencing projects have assumed a central role in research in recent years, starting a scientific revolution. New, non-conventional approaches based on complete genome sequences could help solve old problems.

As an example of these novel approaches, the proteomic profiles of different *T*. *solium* cysticerci were analyzed herein by two-dimensional gels (2D-PAGE) and characterized by bioinformatics.

Our main objective was to identify and characterize the experimental proteome of Mexican *T*. *solium* cysticerci. To achieve this, the supernatants of five cysticercus cultures were analyzed by two-dimensional gels. While similar protein patterns were observed in all cultures, the number and distribution of spots was different for each cysticercus culture. *T*. *solium* is known to release proteins to manipulate the environment in its host, promoting survival, immunomodulation, and pathogenesis. Secreted proteins can be different, depending on the environmental conditions faced by each cyst [28].

To identify the conserved ESPs in cysticercus cultures, the experimental IP and MW values in 2D gels were matched with those predicted in the *T*. *solium* secretome. The conserved proteins thus characterized are interesting in potential therapeutic, immunization, or diagnostic candidates. Additionally, the characterization of differential ESPs among cysticercus cultures could give us insight on possible implications of parasite metabolism for the host.

On the other hand, while the use of 2D-PAGE gels coupled to protein sequencing by MS is a mature and powerful method, widely used for protein characterization, the analysis of a complete proteome is not always possible with this approach. It has been shown that only 40% of the proteins in a typical 2D-PAGE gel are sufficiently abundant to be identified by MS (20).

Therefore, new techniques that allow a large-scale identification of proteins from a complex biological source, independently of their relative expression, are much needed. This study proposes a strategy for protein identification when the conditions for protein sequencing by MS are not met.

Secretion proteins were found in differing amounts in each 2D gel (137 spots on average in five cysticercus cultures). Our strategy allowed us to identify the sequence of 91 proteins (on average in the five cultures) in the *T*. *solium* secretome. These results are consistent with reports on the secretome of other parasites determined with 2D-PAGE and/or MS [29–31]. For example, 200 spots could be identified by 2D-PAGE; among them, 107 proteins were identified by MS [29]. In the case of the proteome of *Schistosoma japonicum*, 101 proteins were identified by nanoscale-LC-MS/MS, 53 of which were predicted in the secretome ([31]. In another work, more than 200 spots were identified by 2D-PAGE in *Ascaris summ* proteome [30].

Two main secretion pathways have been described, the classical pathway, which requires a signal peptide to be attached to the secreted protein, and the ER/Golgi-independent protein secretion pathway, also termed as non-classical secretion pathway. The latter is the preferred mechanism to translocate angiogenic growth factors, inflammatory cytokines, extracellular matrix components that regulate cell differentiation, proliferation and apoptosis, viral proteins, and parasitic surface proteins, presumably involved in host infection [32].

To determine whether a specific secretion pathway was used by *T*. *solium* secreted proteins, a secretion path mapping was performed with two computational tools widely used for this purpose, SignalP and SecretomeP. As shown in Fig 5, 57% of conserved ESPs follow the nonclassical secretion route. Interestingly, 60% of differential ESPs follow the nonclassical pathway as well. This suggests that *T*. *solium* proteins are preferentially secreted through the nonclassical pathway. This could be associated to the production of pathogenic factors.

On the other hand, the conserved and differential proteins in *T*. *solium* cysticercus cultures were functionally characterized by gene ontology annotation with the BlastKOALA tool. The most representative KEGG Orthology identifiers (KOs) both in conserved and differential proteins were those involved in signal transduction. This is not surprising, the process of secretion and transport of molecules is known to depend on second messengers that interact with a molecule on the cell surface, starting a physicochemical process to remodel the extracellular matrix, allowing protein secretion [33].

Interestingly, both conserved and differential proteins in *T*. *solium* cysticercus cultures are involved in common signal transduction pathways (calcium, sphingolipid, cAMP, HIPPO, PI3K/AKT/mTOR). For instance, the HIPPO signaling pathway is involved in restricting cell proliferation, differentiation, and development; it also promotes apoptosis. The main effectors of the HIPPO signaling pathway include the YAP and TAZ transcriptional coactivators, with increased activity in many types of human cancer. Therefore, a deregulation in HIPPO signaling promotes the oncogenic properties of YAP and TAZ [34]. Another signaling pathway present in both data sets is the PI3K/AKT/mTOR pathway, an important intracellular signaling pathway to regulate cell cycle, promote growth and proliferation on the differentiation of adult stem cells, specifically neural stem cells. This way pathway is hyperactive in many types of cancer [35,36]. Interestingly, cancer is another KO highly represented in our data set.

The link of *T*. *solium* infection and cancer is not new, O H Del Brutto (1997) discussed the possible implications between *T*. *solium* pathogeny with the development of brain tumors. Suppressing the host immune response around calcified cysticerci leads to intense astrocytic gliosis, which may contribute to the development of cerebral glioma [37]. A possible association between cysticercosis the appearance of brain tumors has also been described; DNA damage may occur in the cells surrounding cysticerci due to the host chronic inflammatory response. A similar association has been proposed between cysticercosis and the development of hematological malignancies, due to chromosomal aberrations [38].

On the other hand, one of the most overrepresented KOs in the BlastKOALA Brite analysis was found to be linked to exosome synthesis. It is currently known that helminths release exosomes transporting miRNAs and proteins, as another mechanism by which helminths manipulate their host [39]. Exosome-like vesicles containing proteins like enolase have been described in *Taenia asiatica* [40]. Enolase is a glycolytic enzyme widely described in *Taenia* spp. as an ESP. While its primary function is to provide energy for egg production and development, enolase is also involved in host invasion due to its ability to bind plasminogen, favoring the migration of several pathogens [40–43].

This work reports a novel approach to predict the sequence of proteins by their physical-chemical properties. Notwithstanding its promising results, it should be regarded as a preliminary study. The method should be validated in more comprehensive studies including other organisms, to strengthen its advantages and minimize its limitations. One of the main limitations of this approach is the need of a high-quality genome annotation, to reduce the likelihood of prediction errors. Another limitation is the restricted resolution of proteins in 2D-gels; even though this technique allows for resolving up to 10 000 proteins, the most abundant proteins in a sample were found to be overrepresented [44]. Furthermore, the resolution of low-abundance, low-MW, highly hydrophobic proteins with post-transduction modifications is still limited [45].

On the other hand, it should be noted that proteomes are complex, dynamic systems, in which protein expression is influenced by the microenvironment. To date, we only can capture a moment in time. Thus, it is not possible to observe all the proteins that the parasite can secrete, due to the sampling conditions [44].

While it is true that our study has some limitations, it also opens promising perspectives, including the validation of our approach by analyzing other organisms for which the complete genome has been reported, like *Echinococcus granulosus* and the 43 platyhelminths and 153 nematodes in the WormBase ParaSite. That information would allow us to identify proteins that are shared by various parasites and to design diagnostic methods that prevent a cross-reaction between parasites endemic in the same geographic area.

This work demonstrates the potential usefulness of this new method of protein identification in 2D gels to characterize a complex sample even when the conditions for MS sequencing are not met. The only major limitation is that genomic data are required. Identifying these proteins, which have not been characterized due to their low expression, could lead to new therapeutic targets and deepen our understanding of the pathogenesis of *T*. *solium*.

## Supporting information

S1 Fig Theoretical 2D-PAGE gel of conserved proteins match with *Taenia solium* secretome (squares when this protein is found in 2 cysticerci cultures, circles when this protein is found in 3, triangles when this protein is found in 4 and the star when this protein is found in all cysticerci cultures). (TIF)

S1 Table Total 2D-PAGE spots in culture 1 (C1) matching the *Taenia solium* secretome. (PDF)

S2 Table Total 2D-PAGE spots in culture 2 (C2) matching the *Taenia solium* secretome. (PDF)

S3 Table Total 2D-PAGE spots in culture 3 (C3) matching the *Taenia solium* secretome. (PDF)

S4 Table Total 2D-PAGE spots in culture 4 (C4) matching the *Taenia solium* secretome. (PDF)

S5 Table Total 2D-PAGE spots in culture 5 (C5) matching the *Taenia solium* secretome. (PDF)

S6 Table Comparison between MS sequencing and PI/MW sequence prediction. (PDF)
