# Supplementary figures and images for: A novel, sequencing-free strategy for the functional characterization of Taenia solium proteomic fingerprint
Source: PLoS Negl Trop Dis. 2021 Feb 18;15(2):e0009104. doi: 10.1371/journal.pntd.0009104 (PMC7924735; doi:10.1371/journal.pntd.0009104)

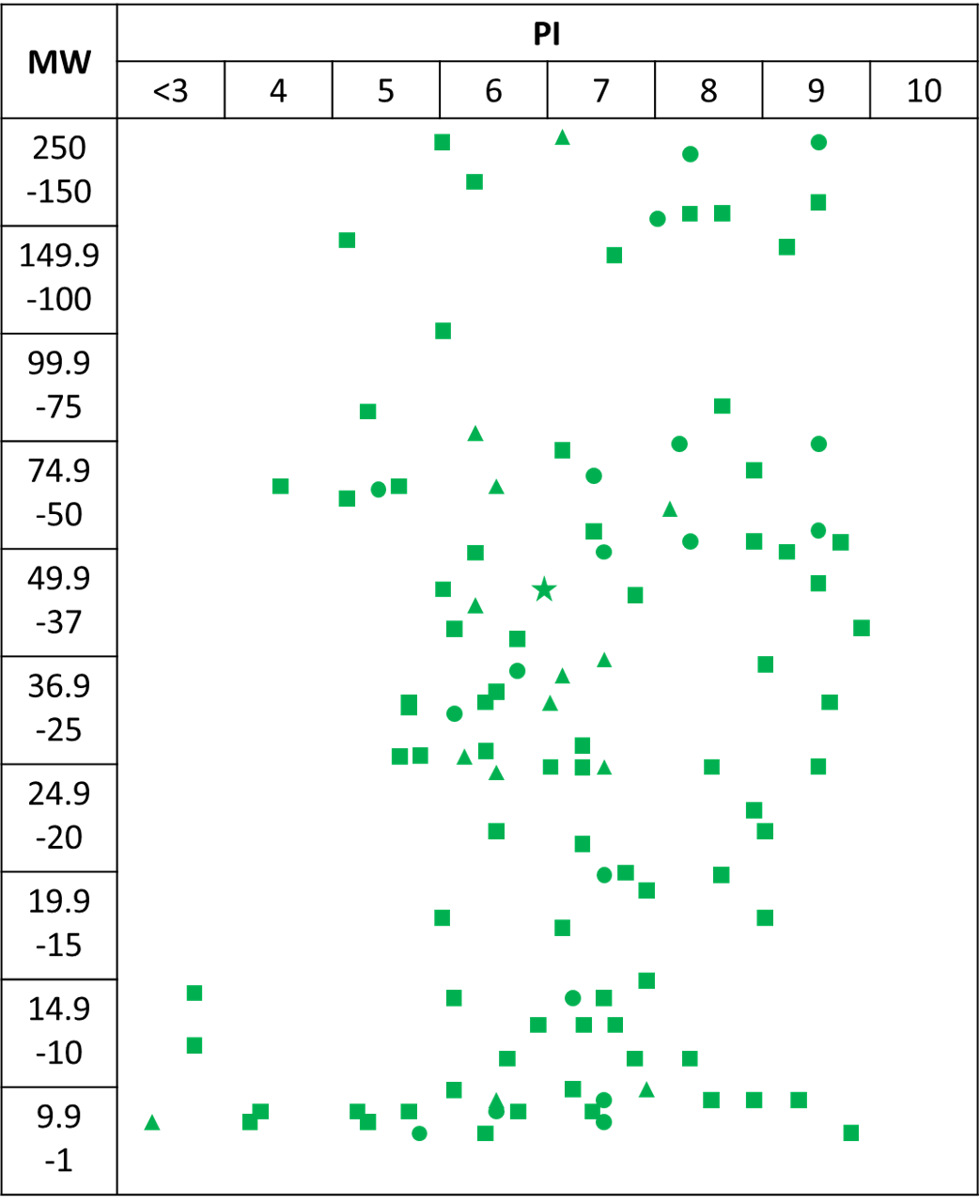

Supplement: S1 Fig — (TIF) [file pntd.0009104.s001.tif]
