# Supplementary material for: A novel, sequencing-free strategy for the functional characterization of Taenia solium proteomic fingerprint
Source: PLoS Negl Trop Dis. 2021 Feb 18;15(2):e0009104. doi: 10.1371/journal.pntd.0009104 (PMC7924735; doi:10.1371/journal.pntd.0009104)
Supplement: S1 Table — (PDF) [file pntd.0009104.s002.pdf]

S1 Table. Total 2D-PAGE spots in culture 1 (C1) matching the *Taenia solium* secretome

Protein ID	IP secretome	MW secretome
TsM_000978800	3.3	7.0
TsM_000437200	3.9	7.6
TsM_000769500	4.3	8.0
TsM_000097200	4.7	8.4
TsM_001239700	5.2	8.1
TsM_000489900	5.5	8.3
TsM_000502600	5.6	22.3
TsM_000794200	5.6	26.1
TsM_000343700	5.7	8.1
TsM_000451800	5.7	16.4
TsM_000706600	5.7	27.8
TsM_000563900	5.7	31.8
TsM_000369000	5.8	6.7
TsM_000990000	5.9	20.0
TsM_000685400	5.9	30.9
TsM_000223900	6.0	18

TsM_000921800	6.1	9.0
TsM_000323600	6.1	14
TsM_000938900	6.1	30.6
TsM_000281800	6.2	26
TsM_000566600	6.3	19.3
TsM_000179800	6.3	21
TsM_000393100	6.3	77
TsM_000253700	6.4	6.2
TsM_000403600	6.4	18.1
TsM_001180100	6.4	32.7
TsM_001016300	6.5	8.0
TsM_000742400	6.5	13.7
TsM_000235300	6.5	16.2
TsM_000264000	6.5	20.6
TsM_000695900	6.5	22.3
TsM_000151800	6.5	24.6
TsM_000771600	6.5	33.0
TsM_000002200	6.5	65.3

TsM_000610500	6.6	21.3
TsM_001066400	6.7	34.5
TsM_000270700	6.8	20.6
TsM_000621600	6.9	8.9
TsM_000132800	6.9	46
TsM_001174200	7.0	31.9
TsM_000163700	7.1	17.1
TsM_000996700	7.1	34.7
TsM_000227000	7.1	77.8
TsM_001007200	7.2	10.7
TsM_000586100	7.2	14.2
TsM_000987700	7.2	273.2
TsM_001046700	7.3	21.3
TsM_001002700	7.3	25.3
TsM_000767500	7.3	27.5
TsM_000254500	7.4	7.8
TsM_000221200	7.4	26.1
TsM_000601600	7.4	67.3

TsM_000344300	7.5	6.7
TsM_000412300	7.5	8.0
TsM_000255700	7.5	9.0
TsM_000342600	7.5	14.2
TsM_000762900	7.5	19.6
TsM_001200300	7.5	25.2
TsM_000985400	7.5	37.9
TsM_000163800	7.5	50.5
TsM_001109200	7.6	13.8
TsM_000790500	7.7	20.7
TsM_001130500	7.8	11.5
TsM_000069900	7.8	44.7
TsM_000991300	7.9	10.1
TsM_000738200	7.9	15.3
TsM_000515400	7.9	19.1
TsM_000938700	8.0	28.8
TsM_000361900	8.0	159.5
TsM_000611600	8.1	40.5

TsM_000902200	8.1	60.1
TsM_000331000	8.2	14.9
TsM_000324100	8.2	15.9
TsM_000001700	8.2	74.9
TsM_000820200	8.3	23.2
TsM_000562700	8.3	51.9
TsM_000428500	8.3	221.8
TsM_001143600	8.4	25.2
TsM_000211600	8.4	206.6
TsM_000951200	8.5	9.0
TsM_001245300	8.5	14.1
TsM_000370900	8.5	24.8
TsM_000109200	8.6	14.4
TsM_000148200	8.6	19.7
TsM_001092400	8.8	10.9
TsM_000729900	8.8	16.1
TsM_000528000	8.9	8.8
TsM_001119700	8.9	12.1

TsM_000079300	8.9	23.4
TsM_000118200	8.9	68.5
TsM_000502100	9.0	17.8
TsM_001067600	9.0	22.0
TsM_000840100	9.1	29.1
TsM_000442400	9.1	61.1
TsM_000233500	9.2	50.1
TsM_001216700	9.3	9.0
TsM_000593700	9.3	19.1
TsM_000416300	9.5	24.9
TsM_000381000	9.5	74.9
TsM_001107700	9.5	232.3
TsM_000804500	9.6	26.4
TsM_000750700	9.7	8.0
TsM_001209200	9.7	51.7
TsM_000193400	9.8	55.0
TsM_000656700	9.9	30.9
TsM_001008100	10.0	7.9

TsM_000954300	10.1	49.9
TsM_000628500	9.1	41.3
