# Supplementary material for: A novel, sequencing-free strategy for the functional characterization of Taenia solium proteomic fingerprint
Source: PLoS Negl Trop Dis. 2021 Feb 18;15(2):e0009104. doi: 10.1371/journal.pntd.0009104 (PMC7924735; doi:10.1371/journal.pntd.0009104)
Supplement: S2 Table — (PDF) [file pntd.0009104.s003.pdf]

S2 Table. Total 2D-PAGE spots in culture 2 (C2) matching the *Taenia solium* secretome.

Protein ID	IP secretome	MW secretome
TsM_000978800	3.3	7
TsM_000629500	3.7	19.3
TsM_000027300	3.8	11.8
TsM_000733700	4.2	13.7
TsM_000413600	4.3	11.3
TsM_000023200	4.4	18.6
TsM_000905900	4.5	18.6
TsM_000370000	4.6	13.7
TsM_000813500	4.7	18
TsM_000430400	5	91.6
TsM_000243100	5	20
TsM_001009400	5.1	138.8
TsM_001100600	5.2	100.5
TsM_000643000	5.3	13.9
TsM_001099800	5.4	98.2
TsM_000095600	5.4	49.6
TsM_000038000	5.4	25.2

TsM_001135300	5.6	112.3
TsM_000627700	5.6	24.2
TsM_001173700	5.6	15.9
TsM_000314700	5.7	31.2
TsM_000057400	6	101.5
TsM_000170000	6	58.3
TsM_000223900	6	17.7
TsM_000590600	6.1	88.4
TsM_000938900	6.1	30.6
TsM_000323600	6.1	14.2
TsM_001133500	6.1	9.7
TsM_000526600	6.2	36.2
TsM_000132000	6.3	195
TsM_000393100	6.3	76.6
TsM_000350600	6.3	43.4
TsM_000982000	6.3	21.8
TsM_001180100	6.4	32.7
TsM_000002200	6.5	65.3
TsM_000771600	6.5	33

TsM_000151800	6.5	24.6
TsM_000695900	6.5	22.3
TsM_000894000	6.5	18.4
TsM_000070800	6.6	118.7
TsM_001111900	6.6	11.2
TsM_000494400	6.7	39.7
TsM_000268600	6.7	38.5
TsM_001066400	6.7	34.5
TsM_000132800	6.9	45.8
TsM_000621600	6.9	8.9
TsM_000236600	7	58.9
TsM_001174200	7	31.9
TsM_000145300	7.1	74.2
TsM_000996700	7.1	34.7
TsM_000163700	7.1	17.1
TsM_000586100	7.2	14.2
TsM_001074900	7.3	14.8
TsM_000238300	7.4	54.1
TsM_000959400	7.4	9.8

TsM_001151600	7.4	9.4
TsM_000163800	7.5	50.5
TsM_000985400	7.5	37.9
TsM_001200300	7.5	25.2
TsM_000762900	7.5	19.6
TsM_001109200	7.6	13.8
TsM_000790500	7.7	20.7
TsM_001130500	7.8	11.5
TsM_000515400	7.9	19.1
TsM_000991300	7.9	10.1
TsM_000361900	8	159.5
TsM_000548600	8	21.4
TsM_000313800	8.1	33
TsM_000001700	8.2	74.9
TsM_000365600	8.3	11.4
TsM_000188800	8.4	19
TsM_000801700	8.5	10.8
TsM_000963100	8.6	83.3
TsM_000439000	8.6	42.5

TsM_000148200	8.6	19.7
TsM_000363800	8.6	14
TsM_000306600	8.6	9.9
TsM_000493400	8.8	75.8
TsM_000066600	8.8	8.8
TsM_000222000	8.9	60.6
TsM_000978300	9	36
TsM_000523800	9	34.4
TsM_000996900	9	25.6
TsM_001067600	9	22
TsM_000502100	9	17.8
TsM_000329600	9	10.8
TsM_001176000	9.1	16.2
TsM_000642200	9.2	23
TsM_000052300	9.2	19.7
TsM_000364700	9.4	22.1
TsM_000925200	9.5	176.3
TsM_000442100	9.5	45.9
TsM_000290900	9.6	116.9

TsM_000741900	9.6	31.1
TsM_001217100	9.7	25.2
TsM_000221800	9.7	8.8
TsM_000547800	9.8	23
TsM_000568600	9.9	40.9
TsM_001227800	6.0	259.3
