# Supplementary material for: A novel, sequencing-free strategy for the functional characterization of Taenia solium proteomic fingerprint
Source: PLoS Negl Trop Dis. 2021 Feb 18;15(2):e0009104. doi: 10.1371/journal.pntd.0009104 (PMC7924735; doi:10.1371/journal.pntd.0009104)
Supplement: S3 Table — (PDF) [file pntd.0009104.s004.pdf]

S3 Table. Total 2D-PAGE spots in culture 3 (C3) matching the *Taenia solium* secretome.

Protein ID	IP secretome	MW secretome
TsM_000676500	4.0	6.6
TsM_000835500	4.5	65.5
TsM_000290400	4.5	4.6
TsM_000792400	4.8	60.9
TsM_000364200	4.8	5.7
TsM_000804700	5.1	62.9
TsM_001231700	5.3	82.1
TsM_000585900	5.3	26.9
TsM_000297800	5.3	6.7
TsM_000478100	5.4	64.3
TsM_000060100	5.6	65.3
TsM_000328200	5.6	8.8
TsM_001226600	5.7	82.4
TsM_000309000	5.8	26.2
TsM_000369000	5.8	6.7
TsM_001220100	5.9	65.5
TsM_000395200	6.0	47

TsM_000439300	6.1	41.2
TsM_000938900	6.1	30.6
TsM_000187700	6.1	24.8
TsM_000765700	6.2	45.5
TsM_000281800	6.2	26.0
TsM_000393100	6.3	77
TsM_000350600	6.3	43
TsM_001246200	6.3	50
TsM_000780800	6.3	29.3
TsM_000430200	6.4	27
TsM_000253700	6.4	6.2
TsM_000002200	6.5	65
TsM_000541900	6.5	28.5
TsM_000151800	6.5	25
TsM_001016300	6.5	8.0
TsM_000464000	6.5	5.2
TsM_001177100	6.6	134
TsM_000494400	6.7	40
TsM_001066400	6.7	35

TsM_000149400	6.7	6.8
TsM_000132800	6.9	45.8
TsM_000328100	6.9	39
TsM_001060800	6.9	13
TsM_000621600	6.9	8.9
TsM_001174200	7.0	31.9
TsM_000179700	7.0	25.9
TsM_000996700	7.1	34.7
TsM_000987700	7.2	273.2
TsM_000767500	7.3	27.5
TsM_001002700	7.3	25.3
TsM_001046700	7.3	21.3
TsM_001154200	7.3	12.8
TsM_000601600	7.4	67.3
TsM_000985400	7.5	37.9
TsM_001200300	7.5	25.2
TsM_000762900	7.5	19.6
TsM_000255700	7.5	9.0
TsM_000344300	7.5	7

TsM_000583000	7.6	8
TsM_000991300	7.9	10.1
TsM_000379500	8.0	42.6
TsM_000902200	8.1	60.1
TsM_000428500	8.3	221.8
TsM_000390400	8.3	167.7
TsM_000674900	8.3	56.5
TsM_000562700	8.3	51.9
TsM_000365600	8.3	11.4
TsM_000350000	8.4	6.5
TsM_000816600	8.5	60.7
TsM_000730500	8.5	51.0
TsM_000389300	8.5	36.9
TsM_000951200	8.5	9.0
TsM_000649300	8.6	165.7
TsM_000963100	8.6	83.3
TsM_000139800	8.7	8.9
TsM_000068200	8.8	59.7
TsM_000376300	8.8	24.6

TsM_000157800	8.9	51.7
TsM_000891900	9.1	64.5
TsM_000098900	9.1	24.8
TsM_000326200	9.2	138.5
TsM_000699700	9.3	11.6
TsM_000852700	9.3	35.0
TsM_000930800	9.3	31.2
TsM_000693700	9.4	29.0
TsM_001107700	9.5	232.3
TsM_000925200	9.5	176.3
TsM_000442100	9.5	45.9
TsM_000384300	9.5	35.1
TsM_000416300	9.5	24.9
TsM_000363000	9.5	4.4
TsM_000193400	9.8	55.0
TsM_000530500	9.8	5.7
