# Supplementary material for: A novel, sequencing-free strategy for the functional characterization of Taenia solium proteomic fingerprint
Source: PLoS Negl Trop Dis. 2021 Feb 18;15(2):e0009104. doi: 10.1371/journal.pntd.0009104 (PMC7924735; doi:10.1371/journal.pntd.0009104)
Supplement: S4 Table — (PDF) [file pntd.0009104.s005.pdf]

S4 Table. Total 2D-PAGE spots in culture 4 (C4) matching the *Taenia solium* secretome.

Protein ID	IP secretome	MW secretome
TsM_000192800	3.3	13.1
TsM_000978800	3.3	7.0
TsM_000629500	3.7	19.3
TsM_000027300	3.8	11.8
TsM_000720300	3.8	7.8
TsM_001185400	4.2	29.9
TsM_001016800	4.2	7.2
TsM_000166100	4.3	45.9
TsM_000029100	4.3	25.2
TsM_001041100	4.3	10.4
TsM_000769500	4.3	8.0
TsM_001234300	4.4	10.0
TsM_000824000	4.5	46.5
TsM_000504000	4.5	29.4
TsM_000580400	4.7	79.3
TsM_000924300	4.7	52.6
TsM_000094700	4.7	48.1

TsM_000380000	4.7	30.6
TsM_001129500	4.7	28.4
TsM_001204400	4.7	26.3
TsM_000484300	4.7	14.6
TsM_000870700	4.9	55.1
TsM_000093800	4.9	34.4
TsM_000794100	4.9	24.9
TsM_001009400	5.1	138.8
TsM_000804700	5.1	62.9
TsM_001097400	5.1	29.2
TsM_000800700	5.2	56.8
TsM_000999900	5.2	49.5
TsM_000671000	5.2	30.7
TsM_001239700	5.2	8.1
TsM_001231700	5.3	82.1
TsM_000807400	5.4	126.8
TsM_000478100	5.4	64.3
TsM_000794200	5.6	26.1
TsM_000983800	5.6	13.8

TsM_000667100	5.7	47.9
TsM_000563900	5.7	31.8
TsM_000314700	5.7	31.2
TsM_000738000	5.7	11.5
TsM_000343700	5.7	8.1
TsM_000351300	5.8	9.6
TsM_000398400	5.9	143.2
TsM_000208300	5.9	40.1
TsM_000057400	6.0	102
TsM_000483700	6.0	55
TsM_000395200	6.0	47
TsM_000439300	6.1	41
TsM_000386100	6.1	8
TsM_000281800	6.2	26
TsM_000132000	6.3	195
TsM_000350600	6.3	43
TsM_000430200	6.4	27
TsM_000756500	6.4	15
TsM_000002200	6.5	65

TsM_001016300	6.5	8
TsM_001111900	6.6	11
TsM_000132800	6.9	46
TsM_001060800	6.9	13
TsM_000621600	6.9	9
TsM_000987700	7.2	273.2
TsM_000586100	7.2	14.2
TsM_001154200	7.3	12.8
TsM_000770200	7.3	8.8
TsM_000601600	7.4	67.3
TsM_000342600	7.5	14.2
TsM_000255700	7.5	9.0
TsM_001161200	7.6	136.6
TsM_000738200	7.9	15.3
TsM_000361900	8.0	159.5
TsM_000902200	8.1	60.1
TsM_000001700	8.2	74.9
TsM_000437600	8.4	8.6
TsM_000370900	8.5	24.8

TsM_000828500	8.6	27.9
TsM_000375000	8.6	12.9
TsM_000225200	9.1	14.2
TsM_001137700	9.1	9.0
TsM_000537100	9.2	10.0
TsM_001216700	9.3	9.0
TsM_000453400	9.4	8.0
TsM_000381000	9.5	74.9
TsM_000740800	9.5	40.3
TsM_000741900	9.6	31.1
