# Supplementary material for: A novel, sequencing-free strategy for the functional characterization of Taenia solium proteomic fingerprint
Source: PLoS Negl Trop Dis. 2021 Feb 18;15(2):e0009104. doi: 10.1371/journal.pntd.0009104 (PMC7924735; doi:10.1371/journal.pntd.0009104)
Supplement: S5 Table — (PDF) [file pntd.0009104.s006.pdf]

S5 Table. Total 2D-PAGE spots in culture 5 (C5) matching the *Taenia solium* secretome.

Protein ID	IP secretome	MW secretome
TsM_000978800	3.3	7.0
TsM_000278800	3.9	7.5
TsM_001016800	4.2	7.2
TsM_000835500	4.5	65.5
TsM_000357000	4.5	7.4
TsM_000502000	4.7	60.2
TsM_000810100	4.8	6.9
TsM_000336600	5.1	72.0
TsM_000297800	5.3	6.7
TsM_000478100	5.4	64.3
TsM_000255000	5.5	61.3
TsM_000060100	5.6	65.3
TsM_000763600	5.6	61.7
TsM_000687200	5.7	50.5
TsM_000374300	5.8	30.2
TsM_000309000	5.8	26.2
TsM_000369000	5.8	6.7

TsM_000826600	5.9	69.7
TsM_000002100	6.0	82
TsM_001133500	6.1	10
TsM_000281800	6.2	26
TsM_000393100	6.3	77
TsM_000740300	6.3	59
TsM_001246200	6.3	50
TsM_000350600	6.3	43
TsM_000151800	6.5	25
TsM_000149400	6.7	7
TsM_000132800	6.9	46
TsM_001174200	7.0	31.9
TsM_000179700	7.0	25.9
TsM_000145300	7.1	74.2
TsM_000996700	7.1	34.7
TsM_000987700	7.2	273.2
TsM_001007200	7.2	10.7
TsM_000885600	7.2	7.9
TsM_000238300	7.4	54.1

TsM_000254500	7.4	7.8
TsM_000163800	7.5	50.5
TsM_000985400	7.5	37.9
TsM_001200300	7.5	25.2
TsM_000219600	7.5	9.4
TsM_000344300	7.5	6.7
TsM_001161200	7.6	136.6
TsM_000069900	7.8	44.7
TsM_000991300	7.9	10.1
TsM_000902200	8.1	60.1
TsM_000428500	8.3	221.8
TsM_000390400	8.3	167.7
TsM_000224800	8.3	70.3
TsM_000562700	8.3	51.9
TsM_000879800	8.4	126.5
TsM_000649300	8.6	165.7
TsM_001070900	8.7	120.3
TsM_000644500	8.7	20.0
TsM_000118200	8.9	68.5

TsM_000157800	8.9	51.7
TsM_000079300	8.9	23.4
TsM_000528000	8.9	8.8
TsM_000978300	9.0	36.3
TsM_000370700	9.0	7.8
TsM_000326200	9.2	138.5
TsM_000233500	9.2	50.1
TsM_000160500	9.2	30.7
TsM_000159300	9.4	58.6
TsM_001107700	9.5	232.3
TsM_000381000	9.5	74.9
TsM_000348600	9.5	72.3
TsM_001209200	9.7	51.7
TsM_000193400	9.8	55.0
TsM_000530500	9.8	5.7
TsM_000568600	9.9	40.9
TsM_000661800	10.0	35.6
TsM_000310900	10.0	7.6
TsM_001227800	6.0	259
