# Supplementary material for: A novel, sequencing-free strategy for the functional characterization of Taenia solium proteomic fingerprint
Source: PLoS Negl Trop Dis. 2021 Feb 18;15(2):e0009104. doi: 10.1371/journal.pntd.0009104 (PMC7924735; doi:10.1371/journal.pntd.0009104)
Supplement: S6 Table — (PDF) [file pntd.0009104.s007.pdf]

S6 Table. Comparison between MS sequencing and PI/MW sequence prediction.

ID (MS sequencing)	Isoelectric point		Molecular weight		ID (secretome)	Culture *Taenia solium*
gi|19879960 8 kDa diagnostic antigen TsRS2 variant 1 [*Taenia solium*]	9.5	9.3	8.7	9.0	TsM_001216700	C1, C4
gi|7339851 immunogenic protein Ts21 [*Taenia solium*]	9.2	9.2	9.7	10.0	TsM_000537100	C4
gi|261266611 enolase [Taenia asiatica]	6.8	6.9	46.7	46	TsM_000132800	C1, C2, C3, C4, C5
gi|311335041 trypsin-like protein [*Taenia solium*]	7.1	7	54.1	58.9	TsM_000236600	C2
gi|4960053 antigen cC1 [*Taenia solium*]	5.7	5.7	38.0	31.8	TsM_000563900	C4, C1
gi|6288722 cysticercosis-specific antigen [*Taenia solium*]	8.9	8.8	9.7	10.9	TsM_001092400	C1
